# Recurrent implantation failure: A comprehensive summary from etiology to treatment

**DOI:** 10.3389/fendo.2022.1061766

**Published:** 2023-01-05

**Authors:** Junying Ma, Wenyan Gao, Da Li

**Affiliations:** ^1^ Center of Reproductive Medicine, Shengjing Hospital of China Medical University, Shenyang, China; ^2^ Key Laboratory of Reproductive and Genetic Medicine, China Medical University, National Health Commission, Shenyang, China; ^3^ Shengjing Hospital of China Medical University, Key Laboratory of Reproductive Dysfunction Diseases and Fertility Remodeling of Liaoning Province, Shenyang, China; ^4^ Department of Obstetrics, the First Affiliated Hospital of China Medical University, Shenyang, China

**Keywords:** recurrent implantation failure, immunology, thrombophilias, endometrial receptivity, microbiome, anatomical abnormalities, aneuploidy

## Abstract

Implantation is the first step in human reproduction. Successful implantation depends on the crosstalk between embryo and endometrium. Recurrent implantation failure (RIF) is a clinical phenomenon characterized by a lack of implantation after the transfer of several embryos and disturbs approximately 10% couples undergoing *in vitro* fertilization and embryo transfer. Despite increasing literature on RIF, there is still no widely accepted definition or standard protocol for the diagnosis and treatment of RIF. Progress in predicting and preventing RIF has been hampered by a lack of widely accepted definitions. Most couples with RIF can become pregnant after clinical intervention. The prognosis for couples with RIF is related to maternal age. RIF can be caused by immunology, thrombophilias, endometrial receptivity, microbiome, anatomical abnormalities, male factors, and embryo aneuploidy. It is important to determine the most possible etiologies, and individualized treatment aimed at the primary cause seems to be an effective method for increasing the implantation rate. Couples with RIF require psychological support and appropriate clinical intervention. Further studies are required to evaluate diagnostic method and he effectiveness of each therapy, and guide clinical treatment.

## 1 Introduction

**Figure 1:**
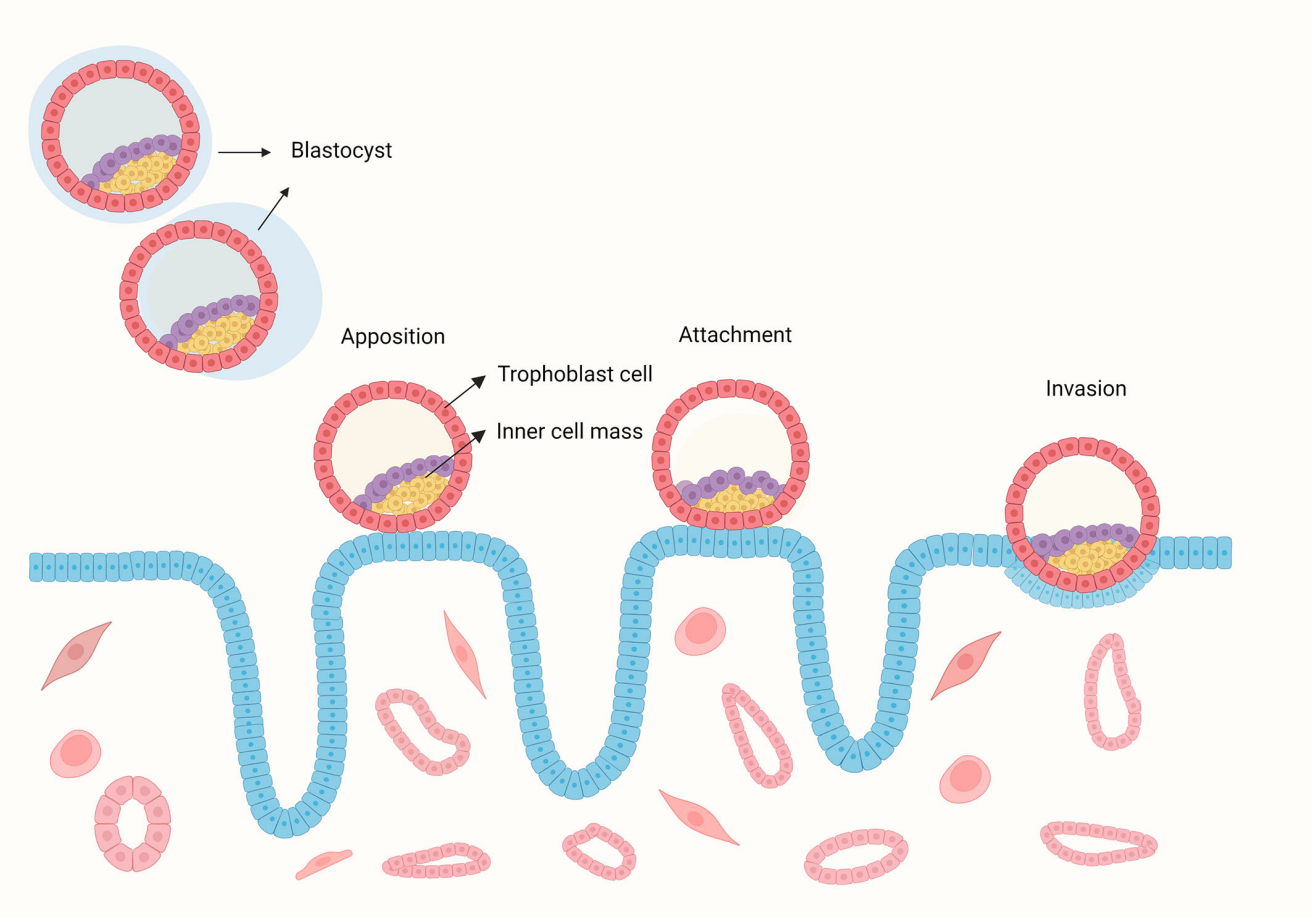
The process of human blastocyst implantation. The blastocyst hatches from the zona pellucida and contact with the endometrium, then the embryo bind to the endometrium during the attachment stage, during with the crosstalk between the embryo and endometrium induces up-regulation of surface receptors and the secretion of signalling molecules and hormones. This signalling directs epithelial withdrawal and trophoblast invading the endometrium.

Implantation is the first step of crosstalk between the embryo and endometrium, which is the key point for a successful pregnancy. The implantation process includes apposition, adhesion, and invasion (**Figure 1**) (1). Successful implantation is identified as an intrauterine gestational sac seen on ultrasonography. Implantation failure may occur during the attachment and migration process, with a negative urine or blood test for human chorionic gonadotropin (hCG) or failure to form an intrauterine gestational sac with positive hCG. Recurrent implantation failure (RIF) is a clinical phenomenon with no widely accepted definition. The key factors that need to be considered while establishing the definition of RIF are the number of embryos transferred or unsuccessful *in vitro* fertilization-embryo transfer (IVF-ET) cycles, the quality of embryos, fresh or frozen embryos, and maternal age, which are disputed points. The increase in the cumulative live birth rate with more IVF-ET cycles showed a progressive decline (2). Other analyses showed that after three IVF-ET cycles, cumulative pregnancy rates did not increase significantly, and the pregnancy rate per cycle tended to decrease after three cycles of unsuccessful treatment (3–5). When RIF was defined as two or more implantation failures, the live birth rate was significantly lower than when RIF was defined as three or more implantation failures, which was considered an excessively increased denominator (6). Hence, a blind increase in IVF-ET cycles may not lead to a successful pregnancy, and we need to set a cut-off point for treatment cycles to recognize patients with RIF. Owing to the different quality of embryos, the number of transferred embryos varies from 3 to 10 or more (7). A good-quality embryo has the proper developmental status according to the day of its development (8). A poor-quality embryo implies that patients need to go through more embryos that are transferred to acquire a successful pregnancy. Another factor that should be considered when defining RIF is maternal age. It is well known that pregnancy rates decrease with maternal age (9); older patients required more cycles of blastocyst transfer to reach the same implantation rate as young women (10). Defining RIF without considering maternal age is meaningless. Based on the above considerations, the widely accepted definition of RIF, as presented by Coughlan, is the failure to achieve a clinical pregnancy after the transfer of at least four good-quality embryos in a minimum of three fresh or frozen cycles in a woman under 40 years of age (11). The preimplantation genetic diagnosis consortium of the European Society of Human Reproduction and Embryology (ESHRE) defined RIF as when more than three good-quality embryo transfers or ten embryos in multiple transfer cycles are performed without achieving a clinical pregnancy (12). In clinical practice, an international survey of clinicians and embryologists showed that the majority defined RIF as failed embryo transfer with three cycles, both fresh and frozen, with no agreement on the cutoff upper age (7).

IVF-ET success rates have improved over the decades due to technical improvements, which affect RIF definition, mainly in the number of embryos transferred. In clinical practice, different centers often adjust their definitions according to their own status (7). Overall, we defined RIF as failure to become clinically pregnant after the transfer of at least three good-quality embryos in three fresh or frozen cycles in women under 40 years of age. Here, a good-quality embryo means day 3 embryo ≥ 8 cells, symmetric, with <10% fragmentation (8), or blastocyst with a grade ≥ 3BB (13). However, further analysis of multiple center clinical data with large sample size is needed to process a more internationally accepted definition of RIF. This review summarizes the etiology of RIF and the current clinical treatment.

## 2 Discussion

### 2.1 Risk factors

Known risk factors for RIF include body mass index (BMI), smoking, alcohol consumption, and stress.

#### 2.1.1 Body mass index

Body mass index is associated with implantation. Obesity affects the female reproductive system. Pre-pregnancy obesity is associated with abnormal menstruation, anovulation, and pregnancy complications (14, 15). In IVF-ET, obese patients tend to have a lower pregnancy rate than normal-weight patients (16). Furthermore, when BMI was ≥ 30 kg/m^2^, patients undergoing IVF-ET had significantly decreased odds of implantation (17). In addition, obesity can alter the markers of uterine receptivity and decidualization, which may contribute to a decrease in the implantation rate in obese patients (18).

#### 2.1.2 Smoking

In women undergoing IVF, it is difficult to assess the amount of smoking owing to inaccurate responses to questionnaires, which makes the association between smoking and IVF uncertain. However, for patients who smoked for > 5 years, smoking was associated with fewer oocytes retrieved, a higher cycle cancellation rate, and a lower implantation rate (19). Meanwhile, for male partners, smoking negatively affects sperm motility and counts and increases sperm DNA damage (20).

#### 2.1.3 Alcohol

Alcohol has a negative effect on pregnancy. In developed countries, alcohol use is a risk factor for stillbirth (21) and can also affect the neurocognitive function of the offspring, such as hyperactivity, impulsivity, and lack of awareness of social cues (22). Therefore, couples trying to conceive are advised to quit drinking before pregnancy (23).

#### 2.1.4 Stress

Cortisol production increases in response to stress, which is believed to be a risk factor for pregnancy. Maternal stress, measured by the level of cortisol, increased the risk of miscarriage by 2.7-fold (24). However, another study showed that stress did not affect the outcomes of patients undergoing the first cycle. Failure of the last IVF cycle leads to a high risk of stress (25).

### 2.2 Etiology of recurrent implantation failure

RIF is a complex clinical phenomenon with several different etilogies, including maternal factors, paternal factors and embryo factor. There may not be one single cause, but several factors working together lead to RIF. Among the etilogies, maternal factors include different aspects. Though a good quality embryo is foundation for successful implantation, the state of mother is also crucial, which we will focus on,

#### 2.2.1 Immunology

Successful implantation is a process of maternal-fetal immune tolerance involving various molecules. Trophoblast invasion can activate the maternal immune response to fetal antigens. Local immune cells at the implantation site in the endometrium, which are activated by the embryos, mediate maternal-fetal immune tolerance and promote placental development. They involve in regulating the differentiation of decidual cell, remodeling uterine vascular, promoting epithelial attachment and regulating immune activation. In this stage, immune cells, including innate lymphocytes, T cells, decidual dendritic cells, and macrophages, are activated, and they are also associated with adverse pregnancy outcomes such as RIF (26).

##### 2.2.1.1 Innate lymphocytes

Innate lymphocytes (ILCs) have been proved to exist in human decidua (27). They are divided into two subtypes: natural killer (NK) cells and non-cytotoxic helper ILCs (ILC1s, ILC2s, and ILC3s) (28). NK cells in the uterus (uNk cells) account for over 70% of all endometrial leukocytes in early pregnancy (28, 29) and possess unique functions that differentiate them from peripheral NK cells. They secrete specific chemokines, express unique cell surface markers, and display a large granule morphology. However, they show poor cytotoxicity because they are unable to polarize granules into the immune synapse (30).

NK cells in the decidua stroma secrete cytokines and express receptors mediating maternal-fetal immunity. uNK cells are not directly cytolytic to fetal extravillous trophoblast (EVT) cells (31). They prompt the low cytotoxicity of uNK cells necessary for semi-allogeneic fetus. Specifically, uNK cells express killer cell immunoglobulin-like receptors (KIRs) that can bind to selectively expressed ligands on EVT, such as human leukocyte antigen-C (HLA-C), human leukocyte antigen-G (HLA-G), and human leukocyte antigen-E (HLA-E) (32, 33). The function of uNK cells depends on the balance between inhibitory and activating receptors (34), as KIR genes are highly polymorphic. Each pregnancy involves different maternal/fetal genetic combinations that deliver activating or inhibitory signals to uNK cells. KIR genes can be grouped into two main haplotypes, A and B (35). The maternal KIR genotype could be AA (inhibition of KIR), AB, or BB (activation of KIR). Trophoblast invasion is regulated by interactions between the maternal KIR and fetal HLA-C. Women with the KIR AA genotype have a higher risk of preeclampsia and other pregnancy-related complications (36). About 78% of patients with more than five unsuccessful IVF treatments or embryo transfers lacked three KIR-activating receptors (2DS1, 2DS3, and 3DS5) (37). Moreover, the KIR genotype of Tel AA combined with the HLA-C2C2 genotype was more prevalent in patients with RIF (p/pcorr. = 0.004/0.012, OR = 2.321) (38). This specific combination of polymorphic KIR and HLA-C genotypes can also affect decidual vascular remodeling (39).

**Figure 2:**
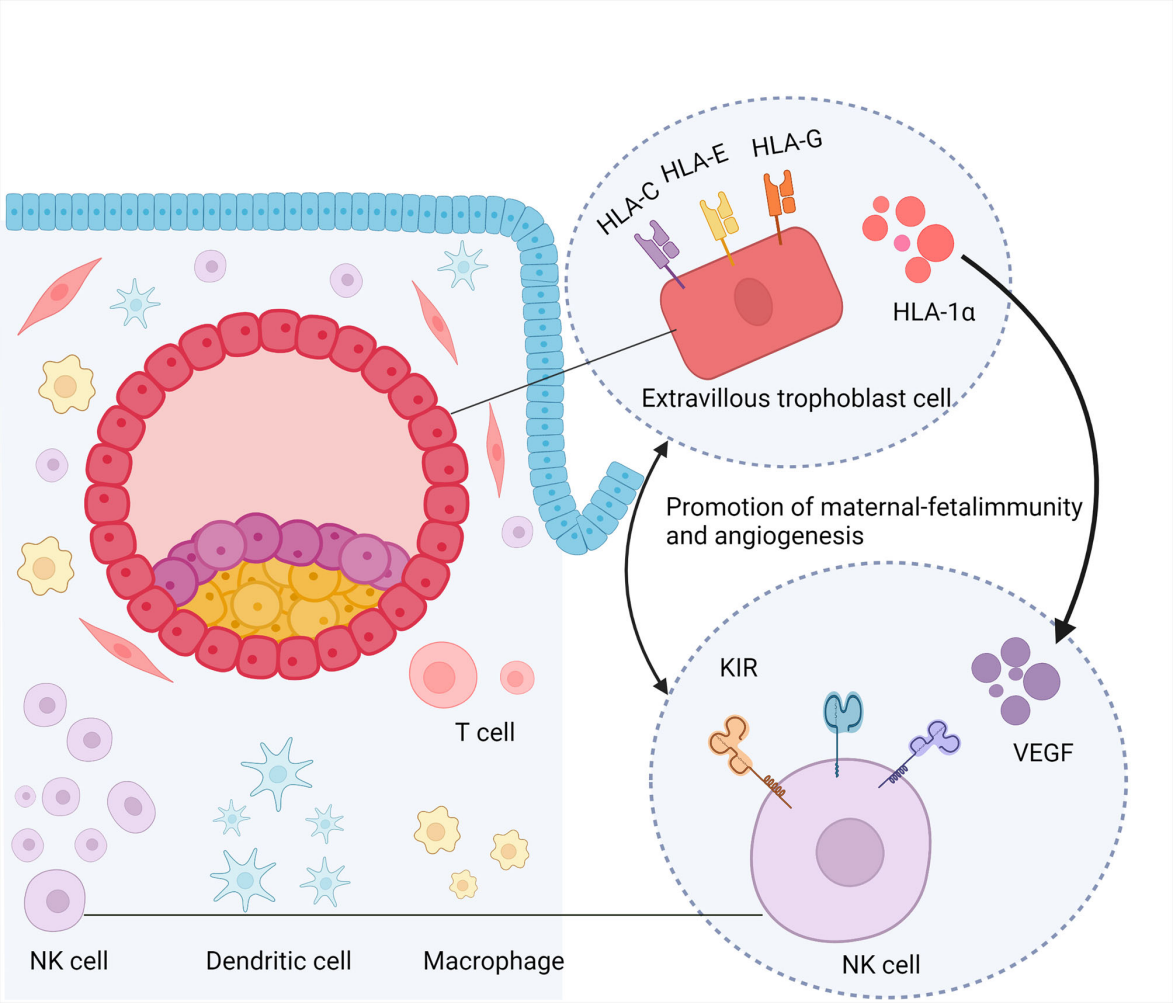
Promotion of maternal-fetal immunity and angiogenesis. NK cell,nature kill cell; DC, dendritic cell; KIR, killer cell immunoglobulin-like receptors; HLA, human leukocyte antigen; VEGF, vascular endothelial growth factor; HIF-1α, hypoxia-inducible factor 1-alpha.

Angiogenesis is the foundation for implantation. uNK cells are the main source of angiogenic growth factors such as placental growth factor, vascular endothelial growth factor (VEGF)-A, and angiopoietin, which may direct angiogenesis during embryo implantation (40, 41). In early pregnancy, uNK cells aggregate around spiral arteries, and animal studies have shown that uNK is involved in spiral artery remodeling (42). These findings suggested that uNK cells play a role in mediating vascular changes during implantation. The number of uNK cells, which was no correlation with peripheral NK level, increases in patients with RIF (43, 44).. However, the production of angiogenic factors, such as VEGF, by uNK cells was lower in patients with RIF than in fertile women, which may be attributed to the increased cytotoxicity of CD16^+^ uNK cells (45, 46). The angiogenic factors produced by uNK cells may be located at the implantation site and move toward the embryo, directing the development of maternal vasculature to the implantation site (47). Hypoxia-inducible factor 1-alpha (HIF-1α) is a transcription factor expressed under hypoxic conditions and can promote angiogenesis by increasing VEGF expression in the tumor tissue. HIF-1α inhibitors can activate the anti-tumor functions of NK cells by elevating interferon-γ (IFN-γ) production (48). In early pregnancy, trophoblasts secrete HIF-1α under hypoxic conditions (49). In the uteri of patients with RIF, both HIF-1α expression and angiogenesis are reduced (50). Therefore, we assume that a decrease in HIF-1α may be involved in RIF *via* the reduction of VEGF secreted by uNK cells or *via* an increase in uNK cell cytotoxicity. This may be due to abnormal interactions between trophoblasts and uNK cells (**Figure 2**). However, further studies are required to support this hypothesis. Other studies suggested that patients with RIF showed more abnormal vascular parameters as estimated by the Doppler test, with more uNK cells producing more IL-12 and IL-18. Dysfunction of cytokine signaling may impair vascular remodeling, leading to excessive or insufficient recruitment of uNK cells (51–53). However, another study reported different conclusions. Analysis of uNK cell numbers using standard immunohistochemistry protocols showed that there was no difference in uNK cell numbers and distribution relative to endometrial arterioles between patients with RIF and women with successful IVF cycles. Furthermore, uNK cell numbers were significantly decreased in women who had successful pregnancies compared with those who did not (54). Overall, uNK cells might impair vascular remodeling *via* abnormal recruitment of NK cells to endometrium, with dysregulated cytokine signaling.

##### 2.2.1.2 T cells

T cells play an important role in immunity during pregnancy. They are divided into four main types: T helper 1 (Th1) cells, Th2, Th17, and regulatory T (Treg) cells. T cells constitute 5–20% of CD45^+^decidual lymphocytes, which display different functions compared to peripheral blood T cells (55). Th2 cell dominance is essential for normal pregnancy (56). An imbalance of Th1/Th2 is associated with reproductive dysfunction. In patients with RIF, the Th1/Th2 ratio increases in the peripheral blood with an increasing Th1 immune response (57). Meanwhile, anti-inflammatory factors, such as IFN-γ and tumor necrosis factor-α (TNF-α), mainly secreted by Th1 cells, were increased in the peripheral blood of patients with RIF (58).

Th17 cells can produce an anti-inflammatory factor, interleukin-17 (IL-17), which promotes the expression of inflammatory mediators. An abnormal Th17 increase in the peripheral blood and decidua is associated with recurrent miscarriages (59–61). In the peripheral blood of patients with RIF, higher numbers of Th17 cells co-exist with exhausted Treg cells (62). Treg cells are known to mediate pregnancy tolerance, and can potently suppress Th1/Th17-mediated immunity (63). More evidence has shown that exhausted Treg cells may lead to adverse pregnancy outcomes; reduced capability of Treg cells to control over-activated T cells may lead to implantation failure (64). The reduced suppressive capability of Treg cells is associated with CD279/PD-1 expression (65), which may play a role in the RIF mechanism. Moreover, intravenous immunoglobulins (IVIG) can improve the implantation rate by increasing Treg cells in the peripheral blood of patients with RIF, verifying the effect of Treg cells in RIF from another side (66).

##### 2.2.1.3 Decidual dendritic cells

DCs account for 10–20% of decidual leukocytes. As antigen-presenting cells, uterine DCs are involved in the recognition of paternal antigens (26). A decrease in immature DCs and an increase in mature DCs were observed in the decidua of women with recurrent spontaneous abortion (67). Few studies have examined the role of DCs in RIF. Depletion of DC in the uterus led to severe impairment of implantation in mice (68). ILT4^+^ DCs were significantly increased in the peripheral blood and endometrium of patients with RIF compared to that in the fertile control group, probably due to the induction of Treg cells (69). Further studies are required to confirm the relationship between DCs and RIF.

##### 2.2.1.4 Macrophages

Macrophages regulate implantation, placentation, fetal development, and vascular remodeling at the maternal-fetal surface (70). Macrophages located close to invade trophoblasts and spiral arteriesto promote implantation during early pregnancy (71). The proportion of uterine macrophages was high in patients with RIF with chronic endometritis and adenomyosis, indicating that macrophages are involved in the pathological process of implantation failure in these patients. However, the underlying mechanism remains unknown (72, 73).

#### 2.2.2 Thrombophilias

Pregnancy is a hypercoagulable condition. Thrombophilias are conditions that predispose individuals to inappropriate blood clot formation (74). Thrombophilia is involved in recurrent pregnancy loss (RPL), but the association between thrombophilia and RIF remains to be elucidated. Thrombophilias are believed to affect implantation by impairing vascularization of the embryo and disturbing blood flow to the decidual vessels (75).

##### 2.2.2.1 Inherited thrombophilias

Inherited thrombophilia commonly refers to a condition in which genetic mutations affect the function or quantity of proteins in the coagulation system (76). The common forms of inherited thrombophilias are genetic mutations in factor V Leiden, proteins S and C, prothrombin, and methylenetetrahydrofolate reductase (MTHFR). Mutations in these genes are increased in women with RPL, including RIF (77–79). Moreover, patients with RIF with thrombophilia most commonly harbor the MTHFR C677T variant, which impairs implantation by disturbing vascularization (78, 79). On the other hand, hyperhomocysteinemia caused by the MTHFR C677T variant is also considered a risk factor for RPL (80).

##### 2.2.2.2 Acquired thrombophilias

The most prevalent acquired thrombophilia is the antiphospholipid syndrome (APS). It is an autoimmune hypercoagulable state diagnosed by the presence of antiphospholipid antibodies, such as anticardiolipin antibodies, lupus anticoagulant antibodies, and/or anti–2-glycoprotein I antibodies. It has been proven that APS is associated with RPL and patients with previous arterial or venous thromboembolic events have a higher risk of pregnancy complications (81). However, the role of APS in RIF remains unclear. Antiphospholipid antibodies can be detected in patients with RIF (82). In a previous study, the frequency of antiphospholipid antibodies inpatients with RIF was significantly higher than that in the fertile group (83). Nevertheless, other studies have not reported an association between APS and RIF. When APS was analyzed in women with a mean of seven failed IVF-ET cycles, there was no significant association between thrombophilias and RIF (84). Therefore, a clinical practice guideline by the Canadian Fertility and Andrology Society does not recommend testing for thrombophilia in patients with RIF (85).

#### 2.2.3 Endometrial receptivity

The endometrium is critical in pregnancy, as it provides an environment for the implantation of developing embryos. Impaired endometrial receptivity is estimated to account for two-thirds of implantation failures (86). Suboptimal endometrial receptivity has been confirmed as a cause of RIF (87). An endometrial biopsy obtained from patients with RIF on the seventh day of progesterone administration revealed 313 genes that were differentially expressed between patients with RIF and the control group (88). Another study revealed differences in several fertility-related genes in cultured endometria of RIF versus patients who became pregnant after IVF-ET (89). Bioinformatical analyses demonstrated that PTGS2, FGB, MUC1, SST, VCAM1, MMP7, ERBB4, FOLR1, and C3 were the key differentia expression genes related to RIF (90). Transcriptomic studies have indicated that patients with RIF express a different endometrial profile compared to the fertile control group on special days of the menstrual cycle. This is assumed to be due to the displacement of the window of implantation (WOI), which affects more than 25% of patients with RIF (87, 91). Furthermore, prostaglandin synthesis appears to be disturbed in patients with RIF and may lead to poor endometrial receptivity (92).

#### 2.2.4 Microbiome and chronic endometritis

The human microbiome, called “the other human genome,” has been involved in normal physiology and homeostasis, associated with states of health and disease (93, 94). The female reproductive tract contains distinct bacterial communities that form a continuous microbiota changing from the vagina to the ovaries (95). Alterations in the vaginal microbiome are involved in female reproductive system diseases such as bacterial vaginosis, urinary tract infections, and also in pregnancy complications (96–98). Hence, we can assume that microbiota might be involved in several steps of IVF-ET, including gametogenesis, implantation, and delivery.

The vagina is dominated by the *Lactobacillus* genus, which has a probiotic influence on the vaginal microenvironment (95). It can inhibit the invasion of bacteria by producing high concentrations of lactic acid and short-chain fatty acids, which maintain the acidic environment of the vagina (99, 100). Infertile women display abnormal vaginal microbiota. *Ureaplasma* spp. in the vagina and *Gardnerella* spp. in the cervix appeared to be related to women with a history of infertility (101). Investigating the vaginal microbiota in patients with unexplained RIF indicated that vaginal *Lactobacillus* (found to be positively correlated with pregnancy rates) was significantly decreased compared to patients who became pregnant in the first frozen embryo transfer (FET) cycle. Patients with RIF presented higher microbial α-diversity than the control group (99). Meanwhile, vaginal *Lactobacillus* in patients with RIF was significantly decreased compared with healthy women, and the vaginal microbiota profiles in patients with RIF had significantly higher levels of five bacterial genera than in healthy women (102). Therefore, the number of vaginal *Lactobacillus* spp. is assumed to be a predictive biomarker of implantation.

The endometrium contains four orders of magnitude fewer bacteriathan the vagina; the vagina harbors approximately 10^10^-10^11^ bacteria (95, 103). High numbers of *Lactobacillus* spp. in the endometrium during the implantation window were associated with higher successful implantation rates, whereas non-*Lactobacillus*-dominated microbiota, such as *Streptococcus*, during the implantation window resulted in negative pregnancy outcomes (104). Bacterial pathogens alter endometrial microbiota, which can result in chronic endometritis. Chronic endometritis is often asymptomatic, leading to inconsistencies in prevalence. The reported prevalence in patients with RIF ranges from 7.7% to 66% (105–109), with a prevalence of 2.8% in patients with general infertility (110). The uterine immune status in chronic endometritis is altered (72). A study on chronic endometritis has shown abundant immune cells in the endometrium and an increase in CD83^+^ mature DCs, CD68^+^ macrophages, CD8^+^ T cells, and Foxp3^+^ Treg cells; these results might be reasonable for impaired endometrial receptivity and recurrent pregnancy failures (72). Furthermore, microbial alterations in chronic endometritis may also disturb immune status by increasing the synthesis of lipopolysaccharide, an important immunomodulator (111).

During pregnancy, the gut microbiota can change in composition or abundance (112). It may also be involved in embryo implantation by affecting the immune system, coagulation system, and endometriosis pathology (113–115). Patients with RIF display abnormal gut microbiota (116), but the relationship between gut microbiota and implantation failure needs to be further investigated.

#### 2.2.5 Anatomical abnormalities

Several types of uterine abnormalities can affect implantation rates, including fibroids, polyps, intrauterine adhesions, Mullerian abnormalities, adenomyosis, and hydrosalpinges. The proportion of unidentified intrauterine abnormalities in patients with RIF varied between 14% and 51% (117–120). Most patients are asymptomatic and remain undiagnosed until they undergo transvaginal ultrasound or hysteroscopy.

##### 2.2.5.1 Uterine fibroids

Fibroids can lead to deformation of the uterine cavity and adhesion, which can prevent the attachment of the embryo to the endometrium. The effect of fibroids on pregnancy outcomes is related to their location. Intramural and subserous fibroids may not have an impact on pregnancy outcomes. Submucosal fibroids can decrease implantation and pregnancy rates in patients undergoing IVF. The mechanism hindering implantation includes increased uterine myometrial contractions, abnormal vascularization, and a disordered cytokine profile (121). A systematic review concluded that patients with submucosal fibroids had lower implantation and live birth rates than the control group. Therefore, the removal of submucosal fibroids before IVF-ET seems to confer benefits (122).

##### 2.2.5.2 Polyps

Polyps in the endometrium are the most frequent uterine lesions in patients with RIF that interfere with embryo implantation (121, 123). They not only affect the deformation of the uterine cavity, but also disturb the implantation process by altering cytokines secreted by the endometrium, such as insulin-like growth factor 1 binding protein and TNF-α (124, 125). The removal of endometrial polyps before intrauterine insemination is believed to improve clinical pregnancy rates (126).

##### 2.2.5.3 Intrauterine adhesion

Intrauterine adhesion often occurs after the curettage of the gravid uterus to terminate the pregnancy. It impairs the functional layer of the endometrium and prevents embryo attachment for successful implantation. A study of 210 patients with RIF who underwent hysteroscopic evaluation showed that the frequency of intrauterine adhesions was 8.5% (127).

##### 2.2.5.4 Mullerian abnormalities

Mullerian abnormalities, such as septate and bicornuate uteri, should be considered in patients with RIF. Compared with other congenital uterine anomalies, partial septate and septate uteri appear to have the poorest reproductive outcomes, such as reduced pregnancy rate, increased risk of first-trimester miscarriage, and preterm birth (128). Among 144 patients undergoing IVF-ET who experienced implantation failure, uterine abnormalities (mainly septate) were found in 14 (9.7%), which led to the assumption that uterine septate may be a factor involved in implantation failure (129). However, a bicornuate uterus is more likely to have less influence on pregnancy. The major risk factors for a bicornuate uterus are mid-trimester abortion and preterm birth (130).

##### 2.2.5.5 Endometriosis

Endometriosis is an estrogen-dependent inflammation with an incidence of up to 50% in infertile women (131, 132). It can affect female IVF-ET in several aspects, including the number of oocytes retrieved, fertilization, and implantation rate (133). The mechanism involves anatomic distortion, oviduct occlusion, abnormal secretion of cytokines involved in endometrial receptivity, and poor oocyte quality (134). In addition, patients with endometriosis-related infertility display different reproductive tract microbiota, which may disturb endometrial receptivity (95). Adenomyosis, defined by the presence of a heterotopic endometrium in the myometrium, is a special form of endometriosis. This can lead to implantation failure in young patients (135). Nevertheless, surgical operation in adenomyosis may not improve clinical outcomes because there is no defined capsule and part of the uterine wall has to be removed (11).

##### 2.2.5.6 Hydrosalpinges

Hydrosalpinges can negatively impact implantation, mainly due to the impairment of embryo development by innutritious fluid (136). Other mechanisms include disturbing endometrial receptivity and physically flushing the embryo out (137). Infertile patients with hydrosalpinges express significantly less αvβ3 integrin, HOXA 10, and leukemia inhibitory factor (LIF) during WOI compared with fertile women (138–140). In IVF-ET, hydrosalpinges are associated with negative outcomes, including lower implantation rates, lower pregnancy rates, and increased spontaneous abortion rates (141, 142). However, the influence of hydrosalpinges on implantation rates appears to be associated with the extent of the hydrosalpinges. One study showed that implantation rates of patients undergoing salpingectomy were not significantly higher than those in the non-intervention group. However, subgroup analysis indicated significantly increased implantation rates when patients with ultrasound-visible hydrosalpinges underwent surgery (143).

#### 2.2.6 Male factors

Although studies have shown that sperm affects early embryogenesis and placental function, the relationship between male factors and RIF remains poorly understood. Sperm DNA damage is related to poor embryo development, and sperm DNA integrity testing is considered to be associated with reproductive failure (144). However, a prospective study with a small number of patients showed that a high DNA fragmentation index was not correlated with RIF (145), which was consistent with another prospective study (146). Therefore, routine testing for DNA fragmentation is not recommended by the American Society for Reproductive Medicine (ASRM) (147). While sperm aneuploidy rates were evaluated by fluorescence *in situ* hybridization techniques, there was a significant increase in the incidence of sex chromosome disomies in patients with a previous history of RIF; however, the implantation rates did not significantly increase in patients who underwent subsequent IVF-ET cycles (148).

In addition, protamines are the largest number of nuclear proteins in human sperm, which are divided into protamine 1 (P1) and protamine 2 (P2). They can package compacted chromatin more efficiently and protect sperm from oxidative damage. Recently, the P1/P2 ratio has been identified as a new parameter of sperm function that can partly predict the fertilization outcome of IVF-ET (149, 150). An abnormal P1/P2 ratio is related to infertility (151). A decreased P1/P2 ratio was associated with poor pregnancy outcomes, including a lower fertilization rate of IVF and a lower implantation rate per embryo in patients undergoing IVF-ET (152). Moreover, the sperm of male partners of women with RPL contained significantly higher P1 and P2, and a lower P1/P2 ratio, indicating that protamines are not only important for fertilization, but also play a role in early embryogenesis (153).

In conclusion, there is insufficient evidence for an association between male factors and RIF. We hypothesized that impaired sperm parameters are more likely to be involved in RIF by affecting the chromosomal constitution of embryos, which will be discussed in the following section.

#### 2.2.7 Embryo factor

Embryos with abnormal chromosomes are recognized as important factors that cause implantation failure or pregnancy loss (154). The probability of chromosomal aneuploidy in embryos also increases with age. In the first trimester, a spontaneous abortion rate as high as 76% has been attributed to chromosomal abnormalities (155).

Chromosomal abnormalities, including translocations, inversions, deletions, and mosaicism, are more common in patients with RIF than in the general population (156). In cleavage embryos, the incidence of complex chromosomal abnormalities, such as three or more abnormal chromosomes, was independent of age but increased in embryos from patients with a history of RIF (157). This complex abnormality is considered mitotically derived because it is more common in embryos than in retrieved oocytes. However, the exact cause of this remains unknown. Furthermore, embryonic mosaicism is the presence of two or more genetically different cell lineages, usually one with an abnormal chromosome and the other with a normal chromosome, and is common in human preimplantation embryos (158, 159). Due to chromosomal abnormalities in this type of embryo, it is reasonable to suspect that mosaicism can influence the implantation rate. Mosaic embryos have lower implantation rates and live births than euploid embryos, and their implantation potential is affected by the extent of mosaicism (160). Typically, embryos with whole-chromosome aneuploidy display negligible implantation potential (161).

### 2.3 Therapy of recurrent implantation failure

The treatment of patients with RIF presents a challenge to clinicians. Various therapeutic options have been proposed to manage RIF, including lifestyle intervention, immunotherapy, anticoagulant, improving endometrium receptivity and sperm quality and preimplantation genetic testing for aneuploidies (PGT-A). Experienced clinicians and embryologists should discuss therapeutic options with patients to address their questions and offer an individualized treatment plan. We discuss below the different interventions that can be used in the management of RIF.

#### 2.3.1 Lifestyle intervention

##### 2.3.1.1 BMI

Patients should be informed that obesity (BMI ≥ 30 kg/m^2^) or underweight (BMI < 19 kg/m^2^) can negatively impact reproduction outcomes. Patients should be advised to return to a normal BMI before IVF-ET treatment. Multidisciplinary approaches include low-energy diets, pharmacotherapy, and bariatric surgery (162). Weight loss before clomiphene treatment in patients with PCOS resulted in improved ovulation and live births (163). Moreover, short-term weight loss before IVF-ET was associated with the retrieval of more metaphase II oocytes (164). For safety, some countries do not allow public funding for IVF-ET treatment in obese infertile patients unless their BMI is within a certain level (165).

##### 2.3.1.2 Smoking

Women planning pregnancy should stop smoking and avoid secondhand smoke for better IVF-ET outcomes (166). Male partners should also abstain from smoking, as smoking increases the production of reactive oxygen species in seminal plasma, alters sperm microRNA content, and increases DNA fragmentation in sperm (167).

##### 2.3.1.3 Alcohol

More than one unit of alcohol per day can reduce the efficiency of IVF-ET, including fertilization and pregnancy rates, and excessive alcohol intake can be harmful to semen quality. Therefore, couples with RIF should reduce alcohol intake to one or two units per week or total abstinence from alcohol before IVF-ET.

##### 2.3.1.4 Stress

Stress is also associated with RIF. Lifestyle interventions such as a healthy diet, regular exercise, and even psychological interventions may reduce psychological distress and improve future IVF-ET outcomes (168).

#### 2.3.2 Optimal IVF-ET procedure

##### 2.3.2.1 Ovarian stimulation protocol

An appropriate controlled ovarian hyperstimulation (COH) protocol should be considered. The stimulation protocol and dose of gonadotrophin require reconsideration if patients have a suboptimal response. Gonadotropin-releasing hormone agonist (GnRHa) combined with human menopausal gonadotropins (HMG) appeared to widen the implantation window compared to a single HMG protocol, resulting in improved IVF-ET success (169). Moreover, the use of long-acting GnRHa for a few months before IVF-ET may increase the pregnancy rate in patients with endometriosis (170). Administration of a single dose of GnRHa in the luteal phase can improve the implantation rate in intracytoplasmic sperm injection (ICSI) cycles (171). This might be partially due to differences in gene expression caused by different luteal support protocols (172). Therefore, it is important to select a specific protocol that includes ovarian stimulation and luteal support in patients with RIF, which may be related to the success rate.

##### 2.3.2.2 Assisted hatching

Assisted hatching (AH) is a technique that includes zona thinning and zona drilling/opening, using chemical, mechanical, or laser energy. The effects of AH remain unclear. Embryos that underwent drilling treatment in frozen/thawed embryo transfer displayed a higher implantation rate but no increase in pregnancy rate (173). A recent meta-analysis showed that it was uncertain of the effect of AH on live birth rates (174). In selected patients such as those with RIF, AH might be beneficial. In patients with RIF older than 38 years, AH caused by partial zona dissection led to a significant increase in implantation and clinical pregnancy rates (175). However, ASRM considered that there is insufficient evidence for the benefit of AH in patients with poor prognosis, including poor-quality embryos, more than two previous IVF-ET failures, and advanced maternal age (176). Generally, considering the influence of AH on the embryo and its controversial effect on RIF, AH should be used cautiously.

##### 2.3.2.3 Embryo transfer

Abnormally elevated estrogen levels in fresh cycles may influence endometrial morphology and receptivity (177). The endometrium in fresh cycles shows a premature secretory phase followed by dyssynchronous stromal and glandular differentiation in the mid-luteal phase (178). Therefore, the implantation rates in fresh embryo transfer were lower than in frozen-thawed cycles (179). Moreover, the embryo transfer stage is important for successful implantation. Implantation rates were higher in the blastocyst transfer group than in the cleavage embryo transfer group in patients with RIF (180). In patients with RIF with a good ovarian response, the implantation rates of fresh cycles were significantly higher in blastocyst transfer; however, the cycle cancellation rates also increased (181). Thus, transfer of blastocysts in frozen-thawed cycles might be a choice for patients with RIF, and sequential cleavage and blastocyst embryo transfer appeared to be beneficial. It can improve clinical pregnancy rates compared to cleavage embryo transfer. Patients with sufficient embryos may attempt this method (182).

#### 2.3.3 Immunotherapy

Maternal-fetal immune tolerance is a necessary condition for successful implantation. Several immunological therapies have been explored to increase implantation rates. Endometrial biopsies and peripheral blood sampling for NK cell type and count or Th cell proportion offer a method to assess the maternal immune status and a rationale for immune-modulating therapies (183).

##### 2.3.3.1 Glucocorticoids

Glucocorticoids are a type of immunomodulator. They can bind to the glucocorticoid receptor on uNK cells and decrease the number of uNK cells (184, 185). A meta-analysis showed that administration of glucocorticoids during routine IVF-ET cycles did not improve live birth rates. However, the use of glucocorticoids in a subgroup of IVF, not ICSI cycles, was related to increasing pregnancy rates with borderline statistical significance, suggesting that specific subgroups of patients might benefit from glucocorticoid therapy (186). Using prednisolone in patients with serum anti-ovarian antibody positivity and at least two previous IVF failures could decrease the serum anti-ovarian antibody level and improve pregnancy outcomes (187). Prednisolone could also improve the implantation rates in patients undergoing ICSI with high-level peripheral CD69^+^ NK cells (188). However, in a selected group of patients (failure to obtain clinical pregnancy after transfer of at least two embryos in at least two fresh or frozen cycles) with elevated uterine NK cells, prednisolone could decrease uNK cell concentration, but with no significant benefit on pregnancy outcomes (185). Therefore, glucocorticoids should be carefully administered to patients with specific indications, the dosage and time are arbitrary.

##### 2.3.3.2 Intravenous immunoglobulin

Intravenous immunoglobulin (IVIG) is produced by the extraction of IgG fractions from the plasma of healthy donors. It can protect the fetus from the maternal immune system by promoting the expansion of suppressor T cells, inhibiting complement deposition, protecting paternal genes by neutralizing anti-HLA antibodies, and reducing the adhesion of T cells to the human placental extracellular matrix (189, 190). In RPL, IVIG is an efficient therapy for improving pregnancy outcomes by affecting the Th1/Th2 ratio and increasing Treg cells (189, 191). Furthermore, IVIG can improve implantation and pregnancy rates in patients with RIF and immune abnormalities (192). The combined application of IVIG, aspirin, and heparin could increase the pregnancy rates and peripheral blood Treg cell proportion in patients with RIF, compared with patients using only aspirin and heparin (66). In addition, IVIG can decrease NK cell percentage and cytotoxicity, and improve pregnancy and live birth rates in patients with reproductive failure (193, 194). IVIG was administered at 200–500 mg/kg body weight (usually 400 mg/kg) 7 days-24 hours before embryo transfer and lasted until fetal pulse detection or every 3 weeks during pregnancy (192).

##### 2.3.3.3 Tacrolimus

Pregnancy is a type of semi-allograft. The maternal immune system treats the fetus as a foreign agent. Excessive immune activation results in implantation failure. Tacrolimus, an immunosuppressant, has been demonstrated to suppress immunological rejection by inhibiting cytotoxic T cell generation, alloantigen-induced lymphocyte proliferation, and the production of IL-2 and IFN-γ (195). It has been used as a plausible treatment for patients with RIF who have an elevated Th1/Th2 ratio and appears to improve pregnancy outcomes (196, 197). However, further evidence is required to support the use of tacrolimus for RIF. Further, its dose and safety need to be carefully assessed.

##### 2.3.3.4 Cyclosporine

Cyclosporine is a typical immunosuppressant that induces immune tolerance in patients with autoimmune diseases and organ transplantation. It can promote the invasion and migration of villous trophoblasts, thereby improving implantation (198). The production of IL-4, a Th2 cytokine, and chemokine CXCL12 is increased by cyclosporine at the maternal-fetal surface (199, 200). Cyclosporine could improve pregnancy outcomes in patients with RPL with an elevated Th1/Th2 ratio in peripheral blood (201). Patients with unexplained RIF receiving cyclosporine treatment since the transfer day showed an obvious improvement in implantation rates, especially of non-high-quality embryos (202). However, in patients with only one unsuccessful transfer cycle of high-quality embryos, cyclosporine treatment did not display benefits for clinical pregnancy outcomes in the following FET cycles (203). Therefore, it is not recommended administration of cyclosporine in RIF patients routinely.

##### 2.3.3.5 Intralipid

Intralipids are fat emulsions containing glycerin, soybean oil, and egg phospholipids, which are used for parenteral nutrition. It can modulate NK cell cytotoxicity and suppress pro-inflammatory cytokine activity (204, 205). In RIF patients with overactivation of NK cells, intralipids can decrease the biomarkers of immune overactivation in the endometrium and increase live birth rates (206). A recent meta-analysis showed that intra-venous intralipid therapy could improve the clinical pregnancy and live birth rates, but the sample sizes of included studies were small, and the treatment protocols were variable (207). However, not all studies showed an improvement after treatment with intralipid. A randomized controlled trial (RCT) in patients with RIF that used 20% (100 mg) intralipid in 500 mL NaCl on the day of embryo transfer demonstrated that the increase in the clinical pregnancy and live birth rates was not significant after intralipid infusion therapy (208). Coulam believed that intralipid is not appropriate for all patients with RIF but for those with some kind of immune abnormality; identifying such patients is essential (209). Overall, there is insufficient evidence regarding the routine use of intralipid therapy in patients with RIF, and a standard treatment protocol is lacking. Large-scale studies are required to explore the effects and safety of intralipids in RIF treatment.

##### 2.3.3.6 Lymphocyte immunization therapy

LIT is an active immunotherapy that can modulate maternal fetal interface immune balance by administering lymphocytes obtained from mother’s partner. It was initially conceived to improve immune tolerance and better for implantation. This immunotherapy was first used to treat RPL, but its current application is controversial. The 2017 ESHRE guidelines for RPL do not recommend the use of LIT in affected patients. Meanwhile, some studies have found LIT to be beneficial for RIF (210, 211), but RCTs analyzing the efficiency of LIT in treating this condition are still lacking. In general, there is insufficient evidence to recommend LIT in patients with RIF, and we should be aware of the possible complications such as infections, autoimmune disorders and formation of irregular antibodies.

#### 2.3.4 Anticoagulants

##### 2.3.4.1 Aspirin

Aspirin is classified as a non-steroidal anti-inflammatory drug. It can inhibit the activity of cyclooxygenase and is, therefore, used as an antithrombotic agent. In terms of reproduction, aspirin contributed to reduce the inflammation in uterus and improve uterine perfusion, which may improve endometrial receptivity (212, 213). Although aspirin can decrease endometrial and uterine arterial blood flow resistance in patients with unexplained RIF (214), no significant differences were found between the aspirin treatment group and control group with respect to implantation and pregnancy rates (215–218).

##### 2.3.4.2 Low molecular weight heparin

LMWH has an activity similar to that of heparin, with an increased half-life and depolymerization. LMWH possess antithrombin or anticoagulation activities. It is speculated that LMWH might prevent placental thrombosis and infarction and modulate decidualization of the endometrium (219, 220). A prospective randomized trial in patients with previous IVF failure and thrombophilia showed a significant increase in implantation and pregnancy rates (221). In RIF patients, LMWH significantly improved live birth rates and reduced miscarriage rates, even though implantation rates were not significantly improved (222). In patients with two or more unexplained failed fresh embryo transfers, LMWH administration from the day after oocyte retrieval led to a tendency of a higher live birth rate with no significant difference, and the implantation rate was also not different (223). Therefore, LMWH may be a potential intervention for patients with RIF, at a dosage of 40mg/day from the day of oocyte retrieval or embryo transfer to 8-12 weeks of gestation.

#### 2.3.5 Endometrial receptivity improvement

##### 2.3.5.1 Intrauterine infusion

###### 2.3.5.1.1 Human chorionic gonadotropin

Generally, hCG can bind to the LH receptor in the endometrium, induce the secretion of cytokines during implantation window and regulate endometrial receptivity and embryo implantation. It is usually administered 0.25–72 hours before embryo transfer at a dosage ranging from 500 to 1000 IU. Administration of hCG appears to regulate embryo implantation among patients with RIF. It can increase the invasion potential of trophoblast cells by modulating the secretion of matrix metalloprotein-2 and tissue inhibitor of metallopeptidase-1 (224). In a previous study, intrauterine injection of hCG before embryo transfer increased the live birth, clinical, and implantation rates of IVF-ET. The effect of 500 IU hCG was better than that of other dosages. However, the outcomes between the first IVF-ET cycle and RIF subgroups did not significantly differ (225). Another study showed that intrauterine injection of hCG before FET improved pregnancy rates in patients with two more implantation failures. Generally, infertile patients may benefit from intrauterine injection of hCG, but further RCTs are needed to confirm these findings.

###### 2.3.5.1.2 Peripheral blood mononuclear cells

PBMCs, such as monocytes and T and B lymphocytes, can induce the secretion of interleukins and growth factors, which appear to be beneficial to the endometrial thickness and receptivity (226). In addition, intrauterine PBMCs can promote embryo attachment and invasion by creating a pathway while moving towards the endometrial stroma (227). The immune cells recruited to the implantation site may not induce initial inflammation for successful implantation in RIF patients, which can be improved by PBMCs (228–230). A recent meta-analysis showed that implantation and live birth rates of patients with RIF were significantly increased in the PBMCs group (227). Another clinical trial confirmed the effect of PBMCs on patients with RIF. Intrauterine infusion of PBMCs before embryo transfer significantly increased implantation rates in frozen-thawed cycles (230). Thus, PBMCs are considered an effective treatment for patients with RIF that lacks initial inflammation (**Table 1**). Blood samples were typically obtained from patients 3 to 5 days before the scheduled embryo transfer, and PBMCs were isolated and cultured with or without hCG, followed by intrauterine infusion. However, larger study populations and more information on the effectiveness and safety of blood products are still needed.

**Table 1:** Researches of treatment options for improving endometrial receptivity of patients with recurrent implantation failure.

Treatment	Year of publication	Number of patients	Age of patients	Number of implantation failure patient experienced	Type of IVF-ET cycle	Approach	Outcomes
**Intrauterine infusion of PBMCs**	2016 (226)	198	Less than 35 years old	Three or more IVF-ET failure	Fresh ET cycle	PBMCs cultured with hCG for 24 h; intrauterine infusion	IR, CPR and LBR improved
	2006 (229)	35	Unknown	Four or more IVF-ET failure	Fresh ET cycle	PBMCs cultured with hCG for 48 h; intrauterine infusion 3 days before fresh ET	IR, CPR, LBR improved
	2017 (230)	216	Unknown	Three or more IVF-ET failure	Frozen/thawed ET cycle	PBMCs cultured with hCG for 24 h; intrauterine infusion day before frozen/thawed ET	IR, CPR, LBR improved in four or more IVF-ET failure group
**Intrauterine infusion** **of PRP**	2022 (231)	85	Age 24 to 52 years old	Unknown	Frozen/thawed ET cycle	Administration of the PRP in day 10-15 of frozen/thawed ET cycles	BPR, CPR and LBR improved, SAR decreased, endometrial thickness increased
	2021 (232)	98	Age 20 to 40 years old	Three or more high-quality frozen-thawed embryo transfers failure	Frozen/thawed ET cycle	Intrauterine infusion of PRP 2 days before frozen/thawed ET	IR, CPR and OPR improved
	2022 (233)	288	Aged 23 to 40 years old	Three or more consecutive implantation failure of at least 6 cleavage-stage embryos or three blastocysts	Frozen/thawed ET cycle	Intrauterine infusion of PRP 2 days before ET	BPR, IR, CPR, LBR improved
**Subcutaneous** **administered G-CSF**	2016 (234)	112	Less than 40 years old	Three or more consecutive implantation failure with three high-grade quality embryos per cycle	Fresh ET cycle	300µg of subcutaneous G-CSF administered 1 h before fresh ET	BPR, IR and CPR improved
	2011 (235)	89	Less than 39 years old	Three previous IVF-ET failure with at least 7 good embryos	Fresh ET cycle	1.5 mg/kg/daily of subcutaneous G-CSF from the day of fresh ET to day of pregnancy test; if positive, continued for 40 days	CPR improved
**Endometrial scratch**	2022 (236)	933	Age 18 to 44 years old	One previous IVF-ET failure	Fresh ET cycle	Endometrial scratch at 5–10 days before the expected menstrual	LBR improved
	2012 (237)	200	Less than 39 years old	Two or more previous ICSI cycle failure	Fresh ET cycle	Endometrial scratch at Day 4-7 in the menstrual cycle before ET cycle	IR, CPR and LBR improved

PBMCs, peripheral blood mononuclear cells; PRP, platelet-rich plasma; G-CSF, granulocyte-colony stimulating factor; IVF-ET, in vitro fertilization-embryo transfer; hCG, human chorionic gonadotropin; IR, implantation rate; CPR, clinical pregnancy rate; LBR, live birth rate; OPR, ongoing pregnancy rate; BPR, biochemical pregnancy rate; SAR, rate of spontaneous abortion; ICSI, intracytoplasmic sperm injection.

###### 2.3.5.1.3 Platelet-rich plasma

PRP is an autologous blood product containing a high concentration of platelets. The release of platelet-derived growth factors, vascular endothelial growth factors, transforming growth factors, and epidermal growth factors from activated platelets results in angiogenesis, cell proliferation, differentiation, and modification of the local immune response (238–241). It can also promote the expression of tissue remodeling genes and reduce fibrosis in mice with Asherman’s syndrome (242). It has been reported that PRP can improve clinical pregnancy rates and endometrium thickness in patients with RIF (231). The benefit of PRP on implantation rates in patients with RIF has been confirmed by other studies (**Table 1**) (232, 233, 243). Thus, PRP is a promising treatment option. In addition, more high-quality and large-scale studies are needed to further assess the effects and safety of PRP.

###### 2.3.5.1.4 Granulocyte colony-stimulating factor

G-CSF is a cytokine produced by endothelial cells, stromal cells, macrophages, and other immune cells (244). It is also produced by decidual cells, which prompted its use as an adjunct treatment (locally or systemically) for patients with a history of RIF or RPL and thin endometrium (85, 245, 246). A meta-analysis showed an increase in clinical rates of intrauterine infusion or subcutaneous injection of G-CSF during both fresh and frozen embryo transfer (247). However, the method of administration and dosage of G-CSF should be carefully selected. G-CSF is typically administered at a dosage ranging from 60 to 300 mg on the day of hCG trigger or embryo transfer. Implantation and pregnancy rates in patients with RIF were not improved by G-CSF intrauterine infusion in two RCTs (248, 249). Furthermore, subcutaneous administration of G-CSF 1 h before fresh embryo transfer resulted in an improvement in clinical pregnancy and implantation rates compared to the control group, which is consistent with the results of other studies (**Table 1**) (234, 235).

##### 2.3.5.2 Endometrial scratch (Biopsy)

Endometrial scratch before implantation appears to cause decidualization and prepares the endometrium for implantation by increasing cytokines such as LIF and IL-11, which are involved in endometrial receptivity (250), and delaying endometrium maturation caused by COH, which might cause synchronization between the endometrium and embryo (251). Moderate-quality evidence has been demonstrated in previous studies. Endometrial scratch on day 7 of the previous cycle and day 7 of the ET cycle appeared to improve the live birth and pregnancy rates in patients with two previous ET cycles, with no evidence of increasing miscarriage rates or bleeding (251). In patients with one previous IVF cycle failure, higher live birth rates were obtained in the endometrial scratch group, with slightly higher expenditures (236). Endometrial scratches performed during hysteroscopy in the cycle preceding ICSI also improved implantation rates in patients with two or more ICSI cycle failures (237). Therefore, this widely used treatment is safe for improving the IVF-ET outcomes. However, the number and degree of injury and the procedure timing need further investigation.

##### 2.3.5.3 Endometrial receptivity assay

An ERA is a transcriptomic analysis of gene expression at different stages of the endometrium that detects WOI and can facilitate “personalized” embryo transfer for every patient. Patients with RIF appeared to have a lower receptivity proportion compared to the control group in the ERA test (74.1% vs. 88%). In RIF patients with a “receptive endometrium” diagnosed by ERA, embryo transfer conducted at the receptivity time led to similar clinical pregnancy rates as in general patients undergoing IVF (87). A 5-year multicenter RCT demonstrated that personalized embryo transfer after ERA diagnosis reached higher implantation and live birth rates at the first embryo transfer cycle in infertile patients (252). Thus, ERA is a unique procedure for endometrial evaluation that can improve endometrium-related implantation failure.

#### 2.3.6 Antibiotics

Antibiotics can cure infections in most patients with chronic endometritis (253). Chronic endometritis is common in patients with RIF, which can be diagnosed and evaluated by hysteroscopy, and the most frequent infectious agents are bacteria and mycoplasmas (109). Patients with RIF and chronic endometritis received oral antibiotic treatment, and the effect was assessed by hysteroscopy with biopsy. In the cure group, a significant increase in pregnancy and live birth rates was reported compared to the group with continuous chronic endometritis after antibiotic treatment (109). A recent meta-analysis also showed that the implantation and clinical pregnancy rates of patients with RIF with cured chronic endometritis were significantly higher than those of patients with continuous chronic endometritis (254). However, different administration routes have led to different results. Intrauterine antibiotic infusion combined with oral antibiotic administration could not improve clinical pregnancy rates, which may be due to the disturbance of intrauterine infusion in the intrauterine environment (255). In general, chronic endometritis is curable in most patients with RIF, which results in a significant increase in the pregnancy outcomes of IVF-ET performed after treatment.

#### 2.3.7 Hysteroscopy

A few patients with normal hysterosalpingogram results show abnormal hysteroscopy findings (127). Hysteroscopy is a valuable diagnostic and treatment tool that can remove small uterine lesions and restore the shape of the uterine cavity in patients with uterine lesions. Correction of the T-shaped uterus was related to high live birth rates and low miscarriage rates in patients with both primary infertility and recurrent miscarriage (256, 257). Although outpatient hysteroscopy did not improve IVF outcomes in patients with RIF with normal ultrasound of the uterine cavity, which may be due to the high proportion of normal uterine cavity (258). For uterine lesions that affect implantation rates, it is necessary to remove them before the next IVF-ET cycle (122, 126, 259, 260).

#### 2.3.8 Male factor

Normal sperm has smooth nuclei with normal chromatin content and head shape. Moreover, severe abnormalities in sperm are related to low fertilization, implantation, and pregnancy rates (261). Intracytoplasmic morphologically selected sperm injection (IMSI) is a non-invasive method that examines sperm under 6000× magnification before injection to obtain optimal sperm. The IMSI procedure before ICSI appears to be beneficial for implantation and clinical rates in patients with repeated IVF-ICSI failure (262). However, other studies did not draw the same conclusions (263). Thus, no specific microscopic criterion exists for evaluating sperm morphology, and more studies are needed to assess the effect of IMSI on IVF-ET outcomes.

#### 2.3.9 Preimplantation genetic testing for aneuploidies

Aneuploidy accounts for implantation failure and early pregnancy loss, as high as 76% in first-trimester spontaneous abortions (155). PGT-A is a technology that can analyze the chromosomes of embryos in IVF-ET and select euploid embryos for subsequent transfer. Single euploid embryos selected by array comparative genomic hybridization were transferred to patients with RIF, which resulted in implantation rates similar to those in the group without RIF (264). Another retrospective cohort study showed the benefit of using PGT-A in patients with RIF and recurrent miscarriage, leading to a significant increase in implantation rates (265). The cumulative implantation rate of patients with RIF who underwent euploid embryo transfer was 95.2%, which means that most RIFs are due to chromosome aneuploidy and can be improved by transferring euploid embryos (266). Therefore, PGT-A appears to be a considerable treatment option for patients with RIF (12). Furthermore, PGT-A should be administered after a careful assessment of the circumstances of each patient. And the influence of mosaicism must be considered.

## 3 Conclusions and future perspectives

RIF remains a complex, growing problem that affects several patients. There are various etiologies, mechanisms, and treatment options (**Table 2**). Identifying the causes of RIF and providing individualized treatment can improve the implantation rate. However, the treatment of RIF remains challenging, and further research on treatment options is needed to assess the potential of each treatment and establish a standard protocol for each patient.

**Table 2:** Summary of etiologies and treatment options of recurrent implantation failure.

		Treatment	Reference
**Risk factors**	Body mass index	Low-energy diets, Pharmacotherapy, and Bariatric surgery	Cheah et al. (2022), Legro et al. (2016)
	Smoking	Stop smoking and avoid secondhand smoke	Fullston et al. (2017), Budani et al. (2017)
	Alcohol	Reduce alcohol intake to one or two units a week or abstinence from alcohol	National Collaborating Centre for Women’s and Children’s Health (UK) (2013)
	Stress	Healthy diet, Regular exercise, Psychological interventions	Frederiksen et al. (2015)
**Maternal factors**	Immunology	Glucocorticoids, Intravenous immunoglobulin, Tacrolimus, Cyclosporine, Intralipids	Forges et al. (2006), Alhalabi et al. (2011), Ahmadi et al. (2017), Abdolmohammadi-Vahid et al. (2019), Nakagawa *er al*. (2015), Cheng et al. (2022), Ledee et al. (2018)
	Thrombophilias	Aspirin, Low molecular weight heparin	Zhang et al. (2022), Potdar et al. (2013),
	Endometrial receptivity	Frozen-thawed embryo transfer, Peripheral blood mononuclear cells, Platelet-rich plasma, Granulocyte colony-stimulating factor, Endometrial scratch, Endometrial receptivity assay	Shapiro *er al*. (2011), Pourmoghadam Z *er al*. (2020), Li *er al*. (2017), Russel et al. (2022), Zamaniyan et al. (2021), Aleyasin et al. (2016), Hou et al. (2021), Shohayeb et al. (2012), Simon et al. (2020)
	Microbiome	Antibiotics	Cicinelli et al. (2015), Vitagliano et al. (2017)
	Anatomical abnormalities	Hysteroscopy and surgery	Garzon et al. (2020), Pritts et al. (2009), Bosteels et al. (2010)
**Male factors**		Intracytoplasmic morphologically selected sperm injection	Shalom-Paz, et al. (2015), Teixeira et al. (2020)
**Embryo factor**		Optimize ovarian stimulation protocol, Assisted hatching, Preimplantation genetic testing for aneuploidies	Stein et al. (1995), Practice Committee of the American Society forReproductive Medicine (2022), Greco et al. (2014), Pirtea et al. (2021)

## Author contributions

JM, DL, and WG performed the literature search and data extraction and played a major role in writing the manuscript. DL critically revised the manuscript. All authors contributed to the article and approved the submitted version.
